# The Expression and Possible Functions of Tenascin-W During Development and Disease

**DOI:** 10.3389/fcell.2019.00053

**Published:** 2019-04-12

**Authors:** Richard P. Tucker, Martin Degen

**Affiliations:** ^1^Department of Cell Biology and Human Anatomy, University of California, Davis, Davis, CA, United States; ^2^Laboratory for Oral Molecular Biology, Department of Orthodontics and Dentofacial Orthopedics, University of Bern, Bern, Switzerland

**Keywords:** tenascin-W, extracellular matrix, development, osteogenesis, stem cells, tumor stroma

## Abstract

Tenascins are a family of multifunctional glycoproteins found in the extracellular matrix of chordates. Two of the tenascins, tenascin-C and tenascin-W, form hexabrachions. In this review, we describe the discovery and domain architecture of tenascin-W, its evolution and patterns of expression during embryogenesis and in tumors, and its effects on cells in culture. In avian and mammalian embryos tenascin-W is primarily expressed at sites of osteogenesis, and in the adult tenascin-W is abundant in certain stem cell niches. In primary cultures of osteoblasts tenascin-W promotes cell migration, the formation of mineralized foci and increases alkaline phosphatase activity. Tenascin-W is also prominent in many solid tumors, yet it is missing from the extracellular matrix of most adult tissues. This makes it a potential candidate for use as a marker of tumor stroma and a target for anti-cancer therapies.

## Introduction

Tenascins are extracellular matrix glycoproteins that have a characteristic domain organization. At the N-terminus tenascins have a region that permits trimerization through coiled–coil interactions, and some tenascins have N-terminal cysteines that can support the covalent linking of two trimers to form a hexabrachion. From N-terminal to C-terminal this multimerization region is followed by one or more epidermal growth factor (EGF)-like domains, a series of fibronectin type III (FNIII) domains, and a fibrinogen-related domain (Chiquet-Ehrismann and Tucker, 2011).

In tetrapods there are four tenascin genes that encode tenascin-C, tenascin-R, tenascin-X, and tenascin-W (Chiquet-Ehrismann and Tucker, 2011). The best studied of these is tenascin-C, which was also the first tenascin to be discovered (Chiquet-Ehrismann and Tucker, 2011; Midwood et al., 2016). Tenascin-C is widely expressed in the embryo at sites of epithelial–mesenchymal interactions and around motile cells, including neural crest cells and migrating neuroblasts and glial precursors. It is also found at sites of branching morphogenesis and in developing smooth muscle, bone and cartilage. In the adult, the expression of tenascin-C is more limited, but it is still expressed at high levels in tendons and in some stem cell niches (Chiquet-Ehrismann et al., 2014). Tenascin-R has a more restricted pattern of expression. It is primarily found around subsets of glia and in perineural nets in the developing and adult central nervous system (Dzyubenko et al., 2016). Tenascin-X is found widely in loose connective tissue in both the late stages of embryonic development and in the adult, often displaying expression that is mostly complementary to that of tenascin-C (Valcourt et al., 2015).

This review will concentrate on the fourth and final member of the tenascin gene family to be identified: tenascin-W. We will summarize its discovery and domain architecture, patterns of expression, effects on cells in culture, relationships to other tenascins, as well as its roles in cancer and potential for translational use.

## Tenascin-W Basics

### Discovery and Domain Organization

Tenascin-W was first identified in the zebrafish (Weber et al., 1998). The “W” is most likely an eponymous reference to the last name of its discoverer. *In situ* hybridization with zebrafish embryos demonstrated that tenascin-W is expressed by migrating sclerotome cells and neural crest cells, together with tenascin-C. The zebrafish tenascin-W described by Weber et al. (1998) has 4 EGF-like domains and 5 FNIII domains, and a predicted molecular weight of 103 kDa. A sixth predicted FNIII domain is found in the genomic sequence of zebrafish tenascin-W which may be available for alternative splicing (Tucker et al., 2006), though alternative splicing of tenascin-W has not been reported.

**FIGURE 1:**
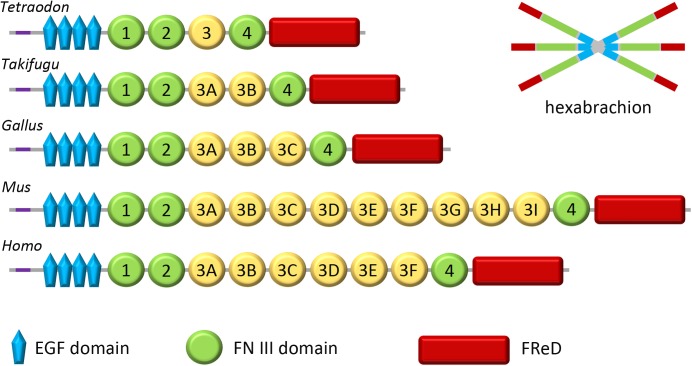
Domain organization of tenascin-W. Tenascin-W has a region near its N-terminus that promotes trimerization (purple); two trimers become covalently linked to form a hexabrachion. Tenascin-W from different species can have different molecular weights as the third FNIII domain (yellow), which is encoded on a single exon, has been duplicated a number of times over the course of evolution. *Tetraodon* and *Takifugu* are genera of pufferfish. *Gallus* is the genus of the chicken and *Mus* is the genus of the mouse. FReD, fibrinogen-related domain.

The homolog of tenascin-W in the mouse was originally named tenascin-N (Neidhardt et al., 2003), which explains why this term and its abbreviation TNN is still encountered in the literature and sequence repositories. The murine homolog was assumed to be a novel tenascin since it has 12 FNIII domains instead of the 5 previously reported in the zebrafish. However, sequence alignment studies reveal that the third FNIII domain of tenascin-W, which is encoded on a single exon, has duplicated repeatedly over the course of evolution (Tucker et al., 2006). Consequently, the number of FN III domains, and hence the size of tenascin-W, can vary significantly from species to species (Figure 1). Synteny also supports the argument that murine tenascin-N is, in fact, tenascin-W: in fish, birds and mammals the tenascin-W gene is found adjacent to the tenascin-R gene (Tucker et al., 2006). In the years that followed the publication of these observations, all peer-reviewed studies focusing on this form of tenascin refer to it as “tenascin-W,” and not tenascin-N. Like tenascin-C, and unlike other tenascins, tenascin-W forms hexabrachions (Scherberich et al., 2004). There is no evidence supporting the formation of tenascin-C/tenascin-W heteromers.

The functions of some of the domains of tenascin-W have been determined experimentally, and potential roles can also be hypothesized from published work with tenascin-C. For example, the FNIII domains appear to contain integrin binding sites (see below), and the fibrinogen-related domains of tenascin-W and tenascin-C share significant similarities and are able to bind and activate Toll-like receptor 4 (Zuliani-Alvarez et al., 2017). Tenascin-W, like tenascin-C, can bind Wnt3a (Hendaoui et al., 2014), but the domain where this binding takes place is unknown. Finally, the EGF-like domains of tenascin-W are nearly identical to those of tenascin-C, and the latter have been shown to be able to activate EGF receptors (Swindle et al., 2001; Fujimoto et al., 2016). Future studies should be directed to see if these properties are unique to tenascin-C, or if tenascin-W may share these and other functional domains with its larger paralog.

### Evolution

While some extracellular matrix molecules like collagens and thrombospondins evolved with the first metazoans (Özbek et al., 2010), others evolved more recently. Tenascins are an example of a family of extracellular matrix molecules that evolved with the first chordates; they are not present in echinoderms, ecdysozoa, or lophotrochozoa (Tucker and Chiquet-Ehrismann, 2009a). A single tenascin gene is found in amphioxus and tunicates, yet neither of these groups has a fibronectin gene (Adams et al., 2015). In lampreys and cartilaginous fish there are two tenascins, and in the latter group these are clearly tenascin-C and tenascin-R. But in bony fish the full complement of four tenascins are seen, including tenascin-W. Thus, tenascin-W apparently evolved together with a bony skeleton, which is intriguing considering its primary sites of expression and its effects on cells *in vitro* (see below).

### Expression at Sites of Osteogenesis and Around Smooth Muscle

**FIGURE 2:**
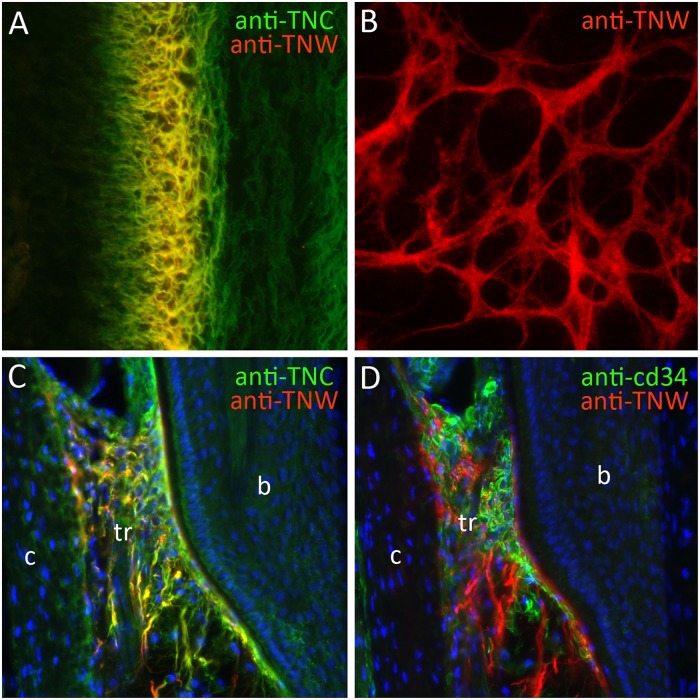
The expression of tenascin-W (TNW). **(A)** Double-label immunohistochemical localization of tenascin-C (TNC) and TNW in the scleral ossicles of an E10 chicken. TNW is commonly expressed at sites of osteogenesis, where it is typically found in the subset of the extracellular matrix labeled with antibodies to TNC. **(B)** After 6 days in culture primary chicken osteoblasts elaborate a TNW-rich extracellular matrix in the presence of BMP2. **(C)** Anti-TNC and anti-TNW both label fibrils in the trabecular region (tr) of adult mouse whisker follicles between the bulge (b) and the capsule (c). TNW-positive fibrils are typically, but not always, a subset of the TNC-positive fibrils. **(D)** The TNW-rich trabecular region contains cd34-positive stem cells.

Following the pioneering study of tenascin-W expression in the zebrafish described above, the expression of tenascin-W was studied in the mouse and chicken. In contrast to tenascin-C, tenascin-W is detected by immunohistochemistry at relatively late stages of development in the chicken. Starting around embryonic day (E) 8, tenascin-W immunostaining is found in areas of osteogenesis and around a subset of smooth muscles in the developing gut (Meloty-Kapella et al., 2006). Later in development it is seen in the cartilaginous skeleton of the heart. Antibodies to tenascin-W label fibrils in the extracellular matrix and it usually, but not always, co-localizes with tenascin-C in these fibrils. Co-localization with tenascin-C is nicely illustrated in developing scleral ossicles in the E10 eye (Figure 2A). The tenascin-W found at sites of osteogenesis likely comes from the osteoblasts themselves, as cultured primary chicken osteoblasts elaborate tenascin-W matrices in the presence of bone morphogenetic protein (BMP) 2 (Figure 2B). The literature lacks, however, detailed descriptions of where tenascin-W is found *in situ*: is it made by osteoblasts only, or is it made by osteoblast precursor cells as well? The latter is possible, and tenascin-W transcripts are detected in MC3T3-E1 preosteoblasts (Schroeder et al., 2007).

During mouse development tenascin-W expression has been studied with both *in situ* hybridization and immunohistochemistry. As in the chicken, tenascin-W in the mouse is expressed at sites of osteogenesis and in subsets of smooth and skeletal muscle during development (Scherberich et al., 2004). It is first seen in the maxillary processes at E11.5. While its expression at sites of osteogenesis typically overlaps extensively with the expression of tenascin-C, its expression in smooth muscle sometimes overlaps and sometimes does not. For example, both tenascin-C and tenascin-W are found in the muscularis mucosa of the stomach at E15.5, but at this stage of development only tenascin-W is found in the muscularis externa. As in the chicken, tenascin-W expression is not observed in the developing nervous system of the mouse (Scherberich et al., 2004). The expression of tenascin-W in a subset of skeletal muscles is evident both from immunohistochemistry (Scherberich et al., 2004) and microarray studies (Fischer et al., 2005). In the mouse, tenascin-W is expressed in developing teeth (Scherberich et al., 2004), and this has been confirmed by microarray analysis of RNA from laser-captured tissues (Sun et al., 2012).

In the adult chicken tenascin-W is detected in the interstitial matrix of the testis and lining brain ventricles (Meloty-Kapella et al., 2006). The latter immunostaining is the only expression of tenascin-W that is related to the nervous system. In the adult mouse tenascin-W expression is observed in periostea, kidney, spleen (Neidhardt et al., 2003; Scherberich et al., 2004) and stem cell niches (see below). Tenascin-W is also seen in some adult human periostea (Tucker et al., 2016).

### Expression in Stem Cell Niches

The first evidence that tenascin-W might play a role in stem cell niches came from studies of its expression in the adult mouse by Scherberich et al. (2004). They observed anti-tenascin-W immunostaining in the corneal limbus and at the base of the cusps of the aortic valves. The limbus niche contains corneal epithelial stem cells (Castro-Muñozledo, 2013), and the cusp base is a potential site of islet1-expressing neural crest-derived stem cells (Engleka et al., 2012). The expression of tenascin-W in bone may also be related to stem cell niches, but this will require more detailed studies for confirmation. Tenascin-W is not detected with immunohistochemistry in the stem cell niche of intestinal crypts (Scherberich et al., 2004; Meloty-Kapella et al., 2006).

One of the most prominent sites of tenascin-W expression in the adult mouse is in hair and whisker follicles (Tucker et al., 2013). In hair follicles tenascin-W is found in a ring just proximal and immediately adjacent to the keratin-15-positive bulge. This region corresponds to the location of nestin-positive stem cells (Amoh et al., 2010) that take part in the cyclic regeneration of hair shafts. In the much larger whisker follicle, tenascin-W-positive fibrils are found in the trabecular region. Tenascin-C is also found in this region, and there is extensive, but not complete, overlap in the tenascin-W and tenascin-C-positive fibrils (Figure 2C). The region inside the whisker follicle capsule where tenascin-W is found is filled with cd34-positive stem cells (Figure 2D). These cells may be mast cell progenitors, or they may be a population of stem cells like those near the keratin-15-positive bulge of hair follicles.

Tenascin-W expression has been found to be up- or downregulated in many studies using microarrays. A search of the 2913 datasets published on the GEO website^1^ found by searching for “TNN and tenascin” (TNN is the genetic shorthand for both tenascin-W and troponins) revealed 33 examples where tenascin-W was consistently up- or downregulated (Supplementary Table S1). Many of these are related to tenascin-W expression in dense connective tissue and in stem cell niches; others are related to tenascin-W in cancer (see below).

## The Cell Biology of Tenascin-W

### Tenascin-W as an Adhesion Modulatory Protein

Adhesion modulation can influence cell motility, proliferation and differentiation. Tenascin-C is a classic adhesion modulatory protein (Chiquet-Ehrismann and Tucker, 2011). *In vitro*, cells can attach to tenascin-C, but they typically fail to spread and form focal adhesions. Similarly, cells that have formed focal adhesions on a substratum coated with fibronectin will lose their focal adhesions if tenascin-C is added to the medium. Tenascin-W also has the potential to act as an adhesion modulatory protein. For example, the myoblast-derived osteogenic mouse cell line C2C12 spread and form focal adhesions and stress fibers on fibronectin, but C2C12 cells remain rounded or stellate and fail to form focal adhesions or stress fibers when cultured in the presence of tenascin-W (Scherberich et al., 2004; Brellier et al., 2012a). Moreover, C2C12 cells remain stellate when cultured on fibronectin if tenascin-W is in the culture medium or if they are cultured on a mixed fibronectin/tenascin-W substratum (Brellier et al., 2012a). Similarly, primary osteoblasts cultured from chicken embryo calvarias spread and form stress fibers on fibronectin but fail to spread on combinations of fibronectin and tenascin-W (Meloty-Kapella et al., 2006). Tenascin-W does not demonstrate adhesion modulation with all cell types. For example, the human breast cancer cell line T47D does not adhere to a tenascin-W-coated substratum, in contrast to dermal fibroblast Detroit551 cells, which attach to human tenascin-W in an integrin β1-dependent manner (Degen et al., 2007). In addition, T47D cells are still able to adhere to a mixed fibronectin/tenascin-W substratum, which is not the case for fibronectin/tenascin-C substrata (Degen et al., 2007).

### Effects of Tenascin-W on Cultured Osteoblasts

Tenascin-W may have evolved in bony fish, and a prominent and phylogenetically conserved site of tenascin-W expression is periosteum and other sites of osteogenesis. One way of studying the effects of tenascin-W on bone maturation is to measure alkaline phosphatase activity in cultured osteoblasts in the presence of tenascin-W. When cultured in dishes coated with 1–10 μg/ml of tenascin-W there is a significant increase in alkaline phosphatase activity in primary calvarial osteoblasts from the chicken (Meloty-Kapella et al., 2008). Interestingly, no increase in activity is seen when higher concentrations of tenascin-W are used to coat the dishes. Over time, cultured primary osteoblasts make mineralized foci called rosettes. In the presence of tenascin-W, significantly more rosettes form. Tenascin-W also influences osteoblast motility in culture. When primary osteoblasts are cultured on tenascin-W-coated filters, significantly more cells migrate across the filter when compared with uncoated filters or filters coated with tenascin-C. Unlike tenascin-C, tenascin-W has no effect on primary osteoblast proliferation.

### Regulation of Tenascin-W Expression

In contrast to the other tenascins relatively little is known about how tenascin-W expression is regulated (Chiovaro et al., 2015a). The UCSC Genome Browser reveals a high degree of conservation between the first non-coding exons of human and murine tenascin-W, as well as in the region extending up to 600 bp 5′ to the first exon. This indicates that there are likely conserved promoters in this region, but more experimental research is clearly needed. This region contains both a TATA box (conserved with tenascin-C) and predicted SMAD-binding elements, and both appear to be active (Chiovaro et al., 2015b). Some potential transcriptional regulators and regulatory pathways are suggested by microarray analyses, including sonic hedgehog, Wilms tumor protein 1, and Notch 1 (Supplementary Table S1). *In vitro*, transforming growth factor (TGF) β1 upregulates tenascin-W in bone marrow stem cells (Chiovaro et al., 2015b), and BMP2 induces tenascin-W in both C2C12 (Scherberich et al., 2004) and HC11 cells (Scherberich et al., 2005).

### Effects of Tenascin-W on Stem Cells *in vitro*

Stem cells can be cultured from their niche in the trabecular region of whisker follicles by stripping the follicle of its capsule, cutting the whisker on either side of the niche, and putting the whisker with the attached niche onto coated plastic dishes. Over time, the stem cells migrate away from the niche and their behavior and morphology can be studied (Sieber-Blum and Hu, 2008). As seen earlier with primary osteoblasts and C2C12 cells, these stem cells will spread and form focal adhesion and stress fibers on fibronectin, but the cells are less spread and have significantly fewer focal adhesions when they migrate onto a mixed fibronectin/tenascin-W substratum (Tucker et al., 2013). The stem cells proliferate at a greater rate in the presence of tenascin-C, but not tenascin-W. What might the role of tenascin-W be in the whisker follicle, and other, stem cell niche? One might be to anchor and concentrate factors like Wnt3a that can influence proliferation and differentiation. Another could be to form a substratum that supports migration: stem cells leaving the trabecular region need to migrate over a millimeter to the whisker follicle bulb, and their pathway is lined with tenascin-W. Insight may come from careful analysis of the tenascin-W knockout mouse, which has yet to be characterized.

### Tenascin-W Signaling

Tenascin-W may be an integrin ligand, and some of its effects on cells in culture may be the results of integrin-based signaling. Scherberich et al. (2004) cultured human T98G glioblastoma-derived cells on murine tenascin-W and assayed the ability of function-blocking antibodies against specific integrin subunits to inhibit this adhesion. The only antibody to block the adhesion was an anti-β1. As a function-blocking anti-α8 was not available, Scherberich et al. (2004) next transfected a cell line that does not normally adhere to tenascin-W with an α8 cDNA, and found that this induced adhesion to tenascin-W. In addition, antibodies to the α8 integrin subunit show expression in many of the same tissues where tenascin-W is found, including periosteum (Scherberich et al., 2004). These observations were expanded upon with 3T3 cells. 3T3 cells normally do not adhere to tenascin-W, but transfecting 3T3 cells with the α8 integrin subunit increases both their adhesion to, and motility on, tenascin-W-coated substrata (Scherberich et al., 2005). The drawback of such studies is that they do not show direct interactions between the ligand and the receptor. α8β1 integrin recognizes the tripeptide motif RGD, and an RGD is found in an exposed loop of the second FNIII domain of mouse tenascin-W. Accordingly, RGD-containing peptides, but not scrambled peptides, block the adhesion of T98G cells to murine tenascin-W-coated substrata (Scherberich et al., 2004). However, an exposed RGD is not found in human or chicken tenascin-W. Interestingly, human and chicken tenascin-C have an RGD motif in the third FNIII, but the third FNIII of mouse tenascin-C does not (Adams et al., 2015). Perhaps tenascin-C and tenascin-W share come integrin-mediated functions and can compensate for each other. Future studies should consider the use of murine tenascin-W with the RGD mutated to another sequence to see if this is, in fact, the sequence recognized by α8β1 integrin.

In summary, studies of cell-tenascin-W interactions *in vitro* indicate that tenascin-W can influence cell adhesion, migration and differentiation, but unlike tenascin-C, tenascin-W does not appear to influence cell proliferation. Tenascin-W seems to modulate cell adhesion in a highly cell type-specific and integrin-dependent way, which might have relevance in the dissemination process of cancer cells during tumor progression (see below).

## Tenascin-W in Cancer

### Tenascin-W Expression in Solid Human Tumors

**FIGURE 3:**
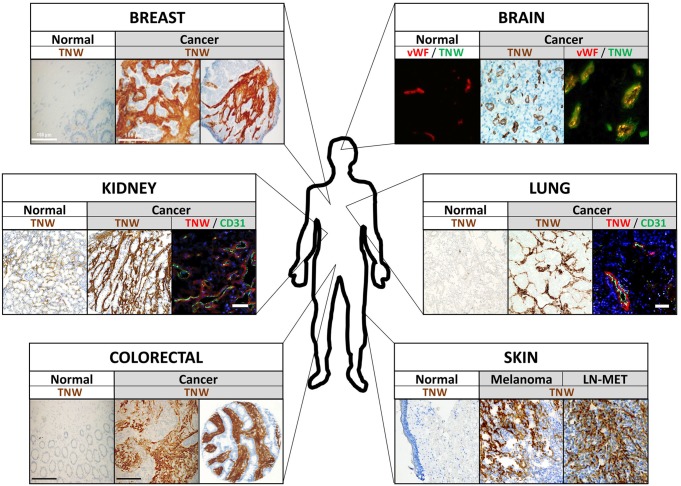
Tenascin-W (TNW) expression in human tumors. Examples of immunohistochemical analyses of TNW in various human tumors and adjacent normal tissue are shown. Note that TNW is prominently expressed in the tumor stroma, often co-localizing with blood vessel markers (see kidney, brain, and lung cancer pictures) and mostly not detectable in the corresponding normal tissue. vWF, von Willebrand factor; cd31, blood vessel marker; LN-MET, lymph node metastasis.

Tumor development has traditionally been viewed as a cell-autonomous process and research was greatly focused on neoplastic cells and the understanding of cancer cell transformation. However, this reductionist vision has changed over the last couple of years. It is now well established that for the full malignant manifestation, tumor cells require a tumor-permissive microenvironment, which is created by a complex interplay between cancer cells and their local environment. Among the proteins known to be specifically expressed in tumor-associated stroma are tenascin-C and tenascin-W. Initially, tenascin-W was found to be overexpressed in the stroma around murine breast tumor lesions having a high likelihood to form metastases (Scherberich et al., 2005). Prompted by these first observations, additional studies on the expression pattern of tenascin-W in human biopsies followed. These analyses revealed complete absence of tenascin-W in most normal adult human tissues and prominent *de novo* expression in all tumors investigated. Tenascin-W is overexpressed in the tumor stroma of breast (Degen et al., 2007), colorectal (Degen et al., 2008), brain (oligodendroglioma, astrocytoma, and glioblastoma) (Martina et al., 2010), kidney (clear cell carcinoma, papillary carcinoma, chromophobe renal carcinoma, and oncocytoma), ovarian, prostate, pancreas, and lung cancers as well as in melanomas (Brellier et al., 2012b; Figure 3). In metastatic melanoma patients, tenascin-W is not only expressed in the primary tumor, but it is also detected in 25% of biopsies received from metastases from diverse locations (i.e., spleen, lung, and skin) as well as in 38% of lymph node metastasis samples (Brellier et al., 2012b). In the tumor stroma, tenascin-W is often found adjacent to blood vessels co-localizing with the endothelial cell markers cd31 and von-Willebrand-factor (Martina et al., 2010; Brellier et al., 2012b; Figure 3).

The cellular source of tenascin-W expression in the tumor stroma remains to be clarified. So far, all available data suggest that tenascin-W expression is restricted to stromal cells. In a xenograft model of breast cancer cells with bone-specific metastatic potential, the bone marrow-derived stromal cells exclusively expressed tenascin-W when co-cultured with the malignant cells (Chiovaro et al., 2015b). Confirming the concept of tenascin-W production by stromal cells, tenascin-W is neither detectable in epithelial-derived cancer cells by immunohistochemistry nor in various human cancer cell lines analyzed and screened by *in vitro* assays. This contrasts with tenascin-C, which can be expressed by stromal as well as cancer cells (e.g., in gliomas) (Sivasankaran et al., 2009).

It is well established that tenascin-C is subject to alternative splicing within its FNIII domains. Certain tenascin-C splice variants containing additional FNIII domains are more tumor-specific than the shortest isoform, and isoform-specific antibodies against tenascin-C can be used as tumor markers. Although tenascin-W shares the modular structure of tenascin-C including a (shorter) stretch of FNIII domains, there is no evidence so far that tenascin-W undergoes alternative splicing in physiological as well tumorigenic conditions.

While tenascin-C and tenascin-W are often co-expressed in the stroma of various solid tumors, certain cancers selectively express only one of the two extracellular matrix proteins (Degen et al., 2007, 2008). These observations suggest the existence of common as well as independent regulatory mechanisms responsible for tenascin-C and tenascin-W expression (Chiquet-Ehrismann and Tucker, 2011). Numerous regulatory mechanisms have been identified for tenascin-C, which include regulation by patterning genes during development, growth factors and inflammatory mediators, hypoxic conditions, mechanical stress, activated signaling pathways, as well as microRNAs (Tucker and Chiquet-Ehrismann, 2009b; Chiquet-Ehrismann and Tucker, 2011; Chiovaro et al., 2015a). Several of these stimuli might be relevant for the tumor-specific expression of tenascin-C. For example, active Notch signaling has been linked to tenascin-C expression during gliomagenesis (Sivasankaran et al., 2009). Knowledge of tenascin-W regulation remains sparse and mostly restricted to mouse and chicken studies. BMP2, BMP7, and tumor necrosis factor (TNF) α induce tenascin-W expression in cultured murine cells, such as mouse embryonic fibroblasts, cranial fibroblasts, C2C12 cells, and HC11 normal mammary gland epithelial cells (Scherberich et al., 2004, 2005; Kimura et al., 2007; d’Amaro et al., 2012) and primary chicken osteoblast cultures. However, this seems to be different in human cells, in which none of the growth factors tested are able to induce endogenous tenascin-W expression. Only recently, TGFβ1 was identified as a first transcriptional regulator of tenascin-W in the metastatic bone marrow niche. *De novo* deposition of tenascin-W by stromal cells was induced via cancer-mesenchymal cell interactions in a TGFβ1-dependent manner (Chiovaro et al., 2015b). Clearly, more research is required for the identification of additional differences (e.g., TGFβ vs. BMPs) or similarities (e.g., negative regulation by glucocorticoids) in the control of *tenascin-C* and *tenascin-W* genes, especially elucidating their abundant *de novo* expression in neoplastic conditions.

### Tenascin-W Promotes Tumor Cell Migration

The first evidence that tenascin-W influences cancer cell migration was provided in transwell migration assays using murine mammary cells plated on inserts coated with mouse tenascin-W. While the normal mouse mammary gland epithelial cell line HC11 failed to migrate across filters coated with tenascin-W, tenascin-W as substrata stimulated the migratory behavior of the highly metastatic 4T1 mouse mammary cancer cell line (Scherberich et al., 2005). This specific response to the presence of tenascin-W was related to the expression of integrin α8, a potential tenascin-W receptor (see above), in 4T1 cells and its absence in HC11 cells (Scherberich et al., 2005). Confirming the motility-promoting activity of tenascin-W, addition of soluble human tenascin-W to the lower side of a transwell migration chamber stimulated cell migration of T47D breast cancer cells toward the fibronectin substratum (Degen et al., 2007). Hence, presence of tenascin-W in the tumor microenvironment stimulates migration of at least breast cancer cells.

### Tenascin-W Promotes Angiogenesis

The observation that tenascin-W co-localizes with endothelial cell markers in the tumor stroma of brain (Martina et al., 2010), kidney, colon, breast, ovary, and prostate cancer (Brellier et al., 2012b), but is completely absent in corresponding normal tissues, encouraged additional studies to determine a possible function of tenascin-W in promoting tumor angiogenesis. Indeed, presence of tenascin-W induces an elongated morphology in human umbilical vein endothelial cells (HUVEC) correlating with increased motility, two characteristic features of angiogenic endothelial cells (Martina et al., 2010). To gain further evidence for a role of tenascin-W in stimulating angiogenesis, a collagen gel-embedded spheroid-based *in vitro* angiogenesis assay was performed. A significant induction of endothelial cell sprouts was observed in HUVEC spheroids embedded in a collagen/tenascin-W gel compared to collagen gels alone (Martina et al., 2010). These data point toward an important role for tenascin-W in stimulating tumor angiogenesis.

### Promotion of Metastasis by Tenascin-W

**FIGURE 4:**
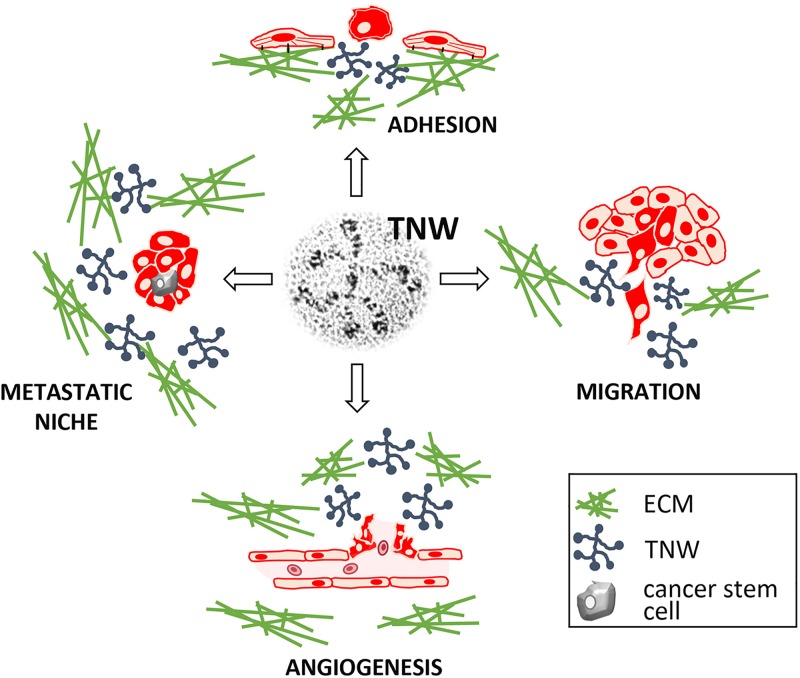
Described functions of tenascin-W (TNW). As a matricellular protein, TNW regulates the interplay between stromal and epithelial cells, and between cells and other extracellular matrix (ECM) components, thereby influencing cancer cell behavior and promoting tumor formation.

Recent data suggest that tenascin-C represents an important component of various physiological as well as pathological stem cell niches, playing fundamental roles in stem cell maintenance and renewal (Chiquet-Ehrismann et al., 2014). Since tenascin-W often shares physiological and tumor-associated expression, as well as functional similarities with tenascin-C, it was not surprising to learn that tenascin-W is present in stem cell niches as well (e.g., corneal limbus and whisker follicles; see above) (Scherberich et al., 2004; Tucker et al., 2013). Similar to tenascin-C, which is part of the lung-metastatic niche for breast cancer cells (Oskarsson et al., 2011), evidence emerged recently that tenascin-W is a component of the congenial metastatic niche in breast cancer cells disseminating to bone (Chiovaro et al., 2015b). After homing of breast cancer cells to bone marrow the malignant cells secrete TGFβ1, which acts in a paracrine fashion to modulate the bone marrow niche, including the *de novo* deposition of tenascin-W. As a specific component of this metastatic niche, tenascin-W promotes metastatic progression by stimulating growth and migration of the cancer cells (Chiovaro et al., 2015b). These data reveal that tenascin-W can be induced in stem cell niches in the bone by breast cancer cell-secreted factors, ultimately leading to an increased risk of bone metastasis. This and other potential roles for tenascin-W in cancer are summarized in Figure 4.

## Potential Translational Use of Tenascin-W

### Cancer Detection

Although tenascin-C and tenascin-W are often overexpressed in tumor stroma, there is now good evidence that the expression of tenascin-W is much more tumor-specific than the expression of tenascin-C (Brellier et al., 2012b). Tenascin-C is readily expressed in certain healthy tissues and it is well established that it can be re-expressed under pathological conditions other than cancers, such as inflammation, healing wounds, infections, and asthma (Orend and Chiquet-Ehrismann, 2006; Brellier et al., 2009; Midwood et al., 2016). Tenascin-W, however, mostly shows no detectable expression in normal tissues adjacent to tumors (Degen et al., 2007, 2008; Brellier et al., 2012b), and so far, there is no *in situ* evidence that it can be induced under conditions other than tumors. For instance, tenascin-W is neither detected in biopsies from patients with inflammatory bowel diseases displaying strong tenascin-C expression (unpublished data) nor in healing wounds in mice (Brellier et al., 2012a). Hence, its prominent and highly tumor-specific expression establish tenascin-W as a potential diagnostic tumor biomarker candidate. Tenascin-W can also be detected in liquid biopsies, making it even more convenient and attractive as a biomarker. Elevated levels of serum tenascin-W have been reported in breast and colon cancer patients compared to controls using a sensitive tenascin-W-specific sandwich ELISA (Degen et al., 2008).

### Prediction of Metastatic Potential

Tenascin-W expression is higher in mouse models of mammary cancer that metastasize compared to non-metastasizing tumors (Scherberich et al., 2005). Up to now, such a correlation was not observed in human breast cancer where tenascin-W expression is enriched in low-grade tumors (Degen et al., 2007). However, in lung cancer, the average expression levels of tenascin-W appear to correlate with tumor grade (Brellier et al., 2012b) and in colorectal cancer, tenascin-W may correlate with the aggressiveness of the disease. Mean serum tenascin-W levels in non-metastatic colorectal cancer patients is 1.5-fold higher in those patients suffering from recurrence compared to non-recurrent patients (Degen et al., 2008). These data suggest that tenascin-W might have potential value as a predictive biomarker. Clearly, these initial studies are too small to draw any conclusive conclusions. Nevertheless, these data warrant more studies to determine the predictive value of tenascin-W in certain malignancies.

### Targeting Therapies

Given its tumor-specific expression and its proximity to blood vessels, we speculate that tenascin-W might be used as a potential candidate for identifying stromal areas with tumors and as a potential target for therapies. Such therapies could include selective delivery of anticancer medicine via tenascin-W specific antibody-drug conjugates. Similar approaches are successfully ongoing with tenascin-C-specific antibodies. In particular, the F16 monoclonal human antibody specifically recognizing the alternatively spliced A1 domain of tenascin-C is being evaluated in clinical trials in combination with approved anti-tumor drugs in patients with solid tumors and metastatic breast cancer (Catania et al., 2015). Since tenascin-W seems to a be a more specific tumor marker than tenascin-C (Brellier et al., 2012b), tenascin-W might represent an additional tumor antigen that could be used as target for antibody-based therapies.

Diverse pro-tumorigenic activities have been attributed to tenascin-W in the tumor stroma. Hence, specifically knocking-down tenascin-W via RNA interference approaches in tumors could interfere with various crucial stages during the malignant progression and could be beneficial for cancer patients. In this regard, it is noteworthy that such strategies are being developed for tenascin-C. Tenascin-C expression was successfully targeted in aggressive brain tumors with a double stranded RNA homologous to tenascin-C triggering its degradation. Glioma patients treated with such an approach benefited from a better quality of life as well from an increase in overall survival. However, the treatment only provided a very short survival advantage (Wyszko et al., 2008).

## Conclusion and Future Directions

Since its discovery in zebrafish 21 years ago (Weber et al., 1998), we have gained significant insight into the structure, evolution, function, and expression of tenascin-W, the final member of the tenascin family. Tenascin-W appears to have evolved together with bone, and a phylogenetically conserved site of tenascin-W expression is at sites of osteogenesis. Tenascin-W is also part of certain adult stem cell niches and is prominent in tumor stroma. In contrast to tenascin-C, tenascin-W is generally absent from normal adult tissue. Cell cultures studies reveal that tenascin-W is an adhesion modulatory protein that can promote bone development, angiogenesis, cell adhesion and cell migration. These observations may be related to the widespread expression of tenascin-W in solid tumors, where tenascin-W may also promote angiogenesis, cell adhesion and cell migration. Whether or not this information will lead to the development of novel cancer diagnosis opportunities or even intervention therapies remains speculative at this point. To make use of tenascin-W as a potential therapeutic entry point we first need to greatly advance our understanding about the impact of the tumor microenvironment on disease progression and about the complexity and multi-functionality of tenascin-W. Compared with tenascin-C, relatively little is known about tenascin-W, but there are some clear directions for future research. The tenascin-W promoter, for example, still needs serious attention. In addition, while the adhesion modulatory properties of tenascin-C have been described in detail, the molecular mechanisms of how tenascin-W affects adhesion remain elusive. For instance, is tenascin-W able to bind to fibronectin, which would be similar to tenascin-C, and which integrins and signaling pathways are involved in regulating the adhesion-modulating characteristics of tenascin-W? Surprisingly, no one has published a characterization of the tenascin-W knockout mouse. This may be partly due to the legacy of the tenascin-C knockout mouse, which was notorious for having a “null phenotype” and the possibility that tenascin-C and tenascin-W might compensate for each other’s absence in certain tissues. However, more detailed studies of the tenascin-C knockout mouse revealed significant defects in behavior that are likely related to abnormal neural stem cell niches, as well as other phenotypes related to abnormal responses to trauma and inflammation. Similarly, detailed studies of the tenascin-W knockout mouse may also prove fruitful.

## Author Contributions

RT wrote the first draft of the Sections “Introduction,” “Tenascin-W Basics,” and “The Cell Biology of Tenascin-W,” and prepared the Figure 1, 2. MD wrote the first draft of Sections “Tenascin-W in Cancer,” “Potential Translational Use of Tenascin-W,” and “Conclusion,” and prepared the Figure 3, 4. Both authors contributed to the final draft and figures.

## Conflict of Interest Statement

The authors declare that the research was conducted in the absence of any commercial or financial relationships that could be construed as a potential conflict of interest.

## Footnotes

1 https://www.ncbi.nlm.nih.gov/geo/
