# Supplementary material for: The Expression and Possible Functions of Tenascin-W During Development and Disease
Source: Front Cell Dev Biol. 2019 Apr 12;7:53. doi: 10.3389/fcell.2019.00053 (PMC6473177; doi:10.3389/fcell.2019.00053)
Supplement: Supplementary file 1 [file Table_1.pdf]

Experiment (Profile)	Expression Trend	Reference
**Dense Connective Tissue**		
Ankle joint response to the induction of arthritis (GDS5244) (GDS5243)	Up	Garcia et al. (2010)
Adaptor protein ASC deficiency effect on bone marrow dendritic cells (GDS5183)	Up	Ippagunta et al. (2011)
MC3T3-E1 preosteoblasts treated with the histone deacetylase inhibitors (GDS3002)	Down	Schroeder et al. (2007)
Pristane-induced arthritis effect on low vs high inflammation models (GDS5052)	Up	De Franco et al. (2014)
Osteoblast differentiation 7 day vs 14 day (GDS1632)	Up	Kalajzik et al. (2005)
Whole embryo vs laser captured tooth germ tissue (GDS4453)	Up	Sun et al. (2012)
**Cancer**		
MCF-7 breast cancer cells treated with laccaic acid A (GDS4972)	Up	Fagan et al. (2013)
Docetaxel resistant breast tumors vs docetaxel sensitive breast tumors (GDS360)	Down	Chang et al. (2005)
**Stem cells**		
Neonatal skin-derived precursors (SKP) and Sox2-positive hair follicle dermal cells (GDS3753)	Up	Biernaskie et al. (2009)
Age effect on hematopoietic stem cells (GDS1803)	Down	Rossi et al. (2005)
Sonic Hedgehog inhibitor cyclopamine effect on co-culture of mesenchymal stem	Down	Rivron et al. (2012)

cells and umbilical vein endothelial cells (GDS4482)		
PLAGL2 effect on primary p53-null neural stem cells (GDS3767)	Down	Zheng et al. (2010)
miR-122 overexpression effect on embryonic stem cells (GDS3470)	Up	Tzur et al. (2008)
Amniotic-fluid kidney progenitor cell-podocytes and conditionally immortalized re-differentiated podocytes (GDS5028)	Down	Da Sacco et al. (2013)
**Respiratory system**		
Lung epithelial cells response to lipopolysaccharide exposure (GDS4253)	Up	Chand et al. (2012)
Claudin 18 deficiency effect on the lung (GDS4961)	Up	Li et al. (2014)
Allergic asthma: bronchial biopsies (GDS4418)	Down	Chamberland et al. (2009)
Age effect on respiratory epithelium (GDS5412)	Up	Wansleeben et al. (2014)
HIV-associated pulmonary arterial hypertension (GDS4229)	Up	Lund et al. (2011)
**Other**		
Notch1 signaling inhibition effect on aortic valve interstitial cells (GDS4488)	Down	Acharya et al. (2011)
Dopaminergic transcription factors Ascl1, Lmx1a, Nurr1 combined effect on embryonic fibroblasts (GDS4155)	Down	Caiazzo et al. (2011)
Teratozoospermia (GDS2695)(GDS2697)	Up	Platts et al. (2007)

IL-10+ vs IL-10- CD8 T cell response to coronavirus infection (GDS4217)	Up	Trandem et al. (2011)
Skin-specific deletion of Scd1 (GDS4910)	Up	Flowers et al. (2011)
Epithelial vs mesenchymal compartments of the developing intestine (GDS2699)	Up	Li et al. (2007)
Lmx1b knockout effect on embryonic proximal hindlimb buds (GDS3320)	Up	Krawchuk and Kania (2008)
Sonic hedgehog homolog-stimulated myofibroblasts (GDS4512)	Down	Chen et al. (2011)
Wilms' tumor protein Wt1 deficiency effect on the urogenital ridge (GDS2747)	Down	Klattig et al. (2007)
SIRT1 deficiency effect on the liver (GDS3666)	Down	Purushotham et al. (2009)
Pancreatic-specific PPAR-beta deletion effect on pancreatic beta-cells (GDS4320)	Down	Iglesias et al. (2012)
Extraocular muscles vs quadriceps femoris (GDS525)	Up	Fischer et al. (2005)
CD40 activation effect on CD19-positive B cells (GDS5407)	Down	Shimabukuro-Vornhagen et al. (2014)
Expression in bladder cells of KLF5 KO mouse (GDS5200)	Down	Bell et al. (2011)

Supplementary Table 1. A search of the GEO Profiles (https://www.ncbi.nlm.nih.gov/gds) with "TNN and tenascin" reveals over 2900 profiles of expression studies including tenascin-W. This table summarizes the trend in tenascin-W expression seen in those published studies (n = 33) that included 5 or more samples with consistent changes of expression seen in each sample when compared with controls. The profiles can be accessed by pasting the profile ID into the search field at the GEO Profiles site.
